# Shear-wave sonoelastographic features of invasive lobular breast cancers

**DOI:** 10.3325/cmj.2016.57.42

**Published:** 2016-02

**Authors:** Boris Brkljačić, Eugen Divjak, Čedna Tomasović-Lončarić, Vanja Tešić, Gordana Ivanac

**Affiliations:** 1 Department of Diagnostic and Interventional Radiology, Breast Unit, University Hospital Dubrava, University of Zagreb School of Medicine, Zagreb, Croatia; 2 Department of Pathology, University Hospital Dubrava, University of Zagreb School of Medicine, Zagreb, Croatia; 3 Department of Epidemiology Institute of Public Health “dr. Andrija Štampar,” Zagreb, Croatia

## Abstract

**Aim:**

To evaluate shear-wave elastographic (SWE) and related gray-scale features of pure invasive lobular breast carcinoma (ILC) and compare them with invasive ductal breast cancers (IDC).

**Methods:**

Quantitative SWE features of mean (El-mean), maximum (El-max), minimum (El-min) elasticity values of the stiffest portion of the mass, and lesion-to-fat elasticity ratio (E-ratio) were measured in 40 patients with pure ILC and compared with 75 patients with IDC. Qualitative gray-scale features of lesion size, echogenicity, orientation, and presence of distal shadowing were determined and compared between the groups.

**Results:**

ILC were significantly larger than IDC (*P* = 0.008) and exhibited significantly higher El-max (*P* = 0.015) and higher El-mean (*P* = 0.008) than IDC. ILC were significantly more often horizontally oriented, while IDC were significantly more often vertically oriented (*P* < 0.001); ILC were significantly more often hyperechoic than IDC (*P* < 0.001). Differences in stiffness between ILC and IDC determined by quantitative SWE parameters were present only in small tumors (≤1.5 cm in size), ie, small ILC had significantly higher El-max (*P* = 0.030), El-mean (*P* = 0.014), and El-min (*P* = 0.045) than small IDC, while tumors larger than 1.5 cm had almost equal stiffness, without significant differences between the groups.

**Conclusion:**

Specific histopathologic features of ILC are translated into their qualitative sonographic and quantitative sonoelastographic appearance, with higher stiffness of small ILC compared to small IDC. Gray-scale and sonoelastographic features may help in diagnosing ILC.

Invasive ductal cancer (IDC) is the most common breast cancer, while invasive lobular cancer (ILC) is the second most common and accounts for 6%-12% of breast cancers (1-3). ILC differs considerably from IDC by having a unique pathological growth pattern, the so called Indian-file pattern, with sheets of single-cell layers growing along the Cooper ligaments, ductuli, and other breast structures, resembling a spiderweb that diffusely spreads in the breast, producing minor desmoplastic reaction (4,5). This spiderweb-like growth is reflected in imaging features of ILC, as well as in its clinical presentation (6). IDC usually clinically manifests as a firm lump, while ILC usually manifests as a palpable thickening and skin or nipple retraction (3,5). ILC has increased tendency for multifocality and multicentricity, a higher risk of bilateral breast cancer (20%-29%), and older age at onset (7,8). Lymph node metastases are less common in ILC than in IDC of equal size, because ILC tumor cells lack cellular atypia and often have low mitotic rate (9). ILC has the propensity to metastasize to the chest, peritoneum, retroperitoneum, and pelvis (10).

Because of its growth pattern of mass infiltrating surrounding tissues, IDC is much more easily detected than ILC also on mammography. ILC has higher false-negative mammographic rates than IDC, since ILC may be invisible or may have quite low mammographic density, and microcalcifications are uncommon (6,11). Due to the higher propensity for multicentric and bilateral lesions, it is generally considered that patients with ILC should be referred to preoperative breast MRI, the best imaging modality to evaluate the tumor extent, while the benefit for preoperative MRI in IDC has not yet been proven (12,13). Fine-needle aspiration is not as sensitive for the diagnosis of ILC as it is for IDC, and core-biopsy should be performed when ILC is suspected, even in cases of palpable lesions (14,15). ILC is associated more often than IDC with positive margins on surgical excision and is more often treated with mastectomy, because of the large size at diagnosis and underestimation of tumor extent with conventional imaging (16).

Ultrasound of the breast is widely used in the diagnosis of breast cancer, usually after mammography, and most image-guided core biopsies of breast lesions are routinely performed under the sonographic guidance (17,18). Ultrasound is highly operator-dependent, much more than mammography or MRI. The quality of ultrasonic equipment and transducers is variable, suboptimal examinations are common, and interobserver variability is high; sensitivity of ultrasound in detection of ILC is reported in the range of 68%-88% (6,12,19).

Sonoelastography is a relatively new ultrasonographic method, which may help in the detection and differentiation of benign and malignant breast lesions (18,20). Strain elastography allows qualitative estimation of the breast lesion stiffness, while shear-wave elastography (SWE) allows quantification of lesion stiffness in kilopascals (kPa) (18). Multicentric studies found that SWE features can help discriminate breast cancers and benign breast lesions, and breast cancers among themselves (20-22). It was also shown that some IDC, like triple negative breast cancers, differ in their stiffness compared to other IDC (23). Studies evaluating some SWE features of invasive cancers were done in a small number of patients with ILC, but to the best of our knowledge none so far has provided values specific for a larger, homogeneous group of patients with pure ILC (24,25).

The aim of this single-center study was to evaluate and establish SWE and related conventional sonographic features of pure ILC of the breast in a group of 40 patients, and to compare these features with the most common invasive breast cancer, IDC. SWE features within ILC group were also correlated with tumor size, extent, histologic grade, and the presence of nodal metastases.

## Materials and methods

The design of this single-center study was retrospective. SWE and related sonographic features were reviewed in 40 female patients with histopathologically confirmed pure ILC, diagnosed in the period of five years (2011-2015) in our department. The same features were analyzed in 75 patients diagnosed with IDC in the period of 18 months (2014 and 2015). The final histopathological diagnosis after surgery was available for all patients. For ILC patients, histopathologic findings were studied for the presence of multifocal disease, angioinvasion, bilateral disease, axillary lymph node metastases, histologic tumor grade, human epidermal growth factor receptor 2 (HER2), and hormone receptor status. Only patients with pure ILC and pure IDC with our without *in-situ* component were included in the study, while the patients with mixed ductulolobular cancers were excluded.

All patients underwent the SWE examination on the same state-of-art ultrasound scanner Aixplorer (Supersonic Imagine, Aix en Provence, France), with the same linear high-frequency 4-15 MHz transducer. All ultrasonographic examinations were performed in the course of the regular diagnostic process and images were taken immediately prior to the ultrasound-guided core biopsy and stored at the hard disk of the ultrasound scanner. Imaging protocol was standardized and all examinations were performed by the single experienced breast radiologist with 25 years experience in breast ultrasound examinations (the first author). The established examination technique was used, without manual compression, with careful electronic focusing of the lesion, and analysis and measurement on B-mode and elastographic image, using always the same preset. The stiffness of the lesion was measured by using the built-in quantification region of interest (ROI) of the system (Q-Box). This is a quantification tool that measures tissue stiffness in kilopascals. ROI size of 2 mm was used in all measurements, placed by the investigator over the stiffest part of the lesion, or at the edge, or within 1-2 mm adjacent to the lesion, at the stiffest part. The stiffest part of the lesion was selected based on the color of the lesion that correlates with kilopascals, and several measurements were performed in the red or bright parts of the lesion, with the highest values recorded. A second ROI of the same size was placed in the fatty tissue of the breast to measure the ratio between mean elasticity values in the lesion and in the fat, the so called elasticity ratio (E ratio).

After generating and capturing a shear wave with ultrafast imaging, the scanner quantifies shear-wave propagation speed and provides the real time SWE color map. Tissue elasticity is derived from the shear-wave propagation speed on the basis of Young’s modulus formula, and the local tissue elasticity values in kilopascals are displayed on the image in a color map along with conventional B-mode image (20,22). The breast preset in the penetration mode was used for all measurements, with the highest stiffness set at ≥180 kPa (7.7 m/s). Quantitative SWE features were measured: mean (El mean), maximum (El max), and minimum (El min) elasticity value of the stiffest portion of the mass, and E ratio (20,22).

Before elastographic measurements, the related sonographic features on the image were analyzed. Only the largest mass/lesion (index lesion) detected on ultrasound per patient was measured, its orientation estimated, and the presence of distal acoustic phenomena (shadowing or enhancement) noted, after which SWE analysis of the lesion was performed. On the gray scale image, the longest diameter of the lesion was measured, and qualitative features, such as echogenicity, orientation of the lesion, and the presence of distal acoustic phenomena, were analyzed. Regarding echogenicity, the lesions were categorized as hypoechoic (if the whole or majority of the lesion had lower echogenicity than the breast parenchyma) or hyperechoic (if the lesion completely or predominantly had higher echogenicity than the parenchyma). Regarding the orientation, lesions were categorized as vertically oriented (when anteroposterior diameter was larger or equal to laterolateral diameter) or horizontally oriented (when laterolateral diameter was larger than anteroposterior diameters). Also, the presence or the absence of the distal acoustic shadowing was noted, while none of the patients exhibited distal acoustic enhancement. The size estimated by ultrasound was used for statistical analysis, because the study evaluated the sonographic appearance of breast cancers. In case of multifocal or bilateral cancers, common in ILC, only the largest, single index lesion per patient was used for SWE evaluation and comparisons between the patient groups. Virtually all lesions had sonographically irregular borders. All images were stored digitally.

Mastectomy or breast conserving surgery was performed for all breast cancers, and the histological type of invasive cancer was available for all patients, determined on the basis of surgically excised specimens. For invasive lobular cancers, data were analyzed for each patient regarding the histological grade, determined using the method of Elston and Ellis, lymph node status, presence of multifocal or bilateral disease, and lymph node status (26). Estrogen receptor (ER), progesterone receptor (PR), and HER2 status were evaluated by immunohistochemistry staining methods according to American Society of Clinical Oncology (ASCO) and College of American Pathologists (CAP) guideline recommendations for HER2 and ER/PR testing. E-cadherin was tested in all patients (27). Sentinel lymph-node biopsy was performed in all patients, followed by axillary dissection in positive cases. Informed consent for ultrasound-guided core biopsy, prior to which sonographic and elastographic analysis was performed, was obtained from patients according to the institutional rules. The study was approved by the institutional ethics committee.

### Statistical analysis

Frequency distributions of selected patients and tumor characteristics were determined for all women. Quantitative variables were tested for normality of the distribution with Kolmogorov-Smirnov test. Differences in lesion size and depth; El mean, El max, and El min values; and E-ratios between patients with IDC and ILC were evaluated using the *t* test. Differences in sonographic features; echogenicity, orientation of the lesion, presence of distal acoustic phenomena; and tumor characteristics were evaluated using the Pearson χ^2^ square exact test. The receiver operating characteristic (ROC) curve analysis was expressed as area under curve (AUC) with its 95% confidence intervals (CI), and the curves were constructed for the El mean, El max, and El min elasticity values; and E-ratios. Diagnostic efficacy for elastographic values was assessed through sensitivity and specificity at cut-off points. All statistical calculations were performed using STATA/IC ver.11.1. (Stata Statistical Software: Release 11. StataCorp LP, College Station, TX, USA). Results of statistical tests with *P* values lower than 0.05 were considered significant.

## Results

The mean age of the patients in the ILC group was 62.2 ± 11.61 years (range, 41 to 91), and of the patients in the IDC group was 60.73 ± 12.61 years (range, 31 to 81). There was no significant difference between the groups in age (*P* = 0.540).

The mean size of index lesions measured by ultrasound was significantly larger in the ILC than in the IDC group (2.25 cm ±1.30 vs 1.55 ± 0.90, *P* = 0.008). Significant differences were also observed in orientation and echogenicity of lesions. Significantly more lesions from the ILC group (31 out of 40, 77.5%) had horizontal orientation (*P* = 0.001), and significantly more lesions from the IDC group (43 of 75, 57.3%) had vertical orientation (*P* = 0.024). Significantly more lesions from the IDC group were hypoechoic (69 of 75, 92.0%), and in the ILC group 13 of 40 lesions (32.5%) were hyperechoic and 27 of 40 (67.5%) were hypoechoic (*P* = 0.001). There was no significant difference in the presence of distal acoustic shadowing, 58.7% (44 of 75) of IDC and 57.5% (23 of 40) of ILC (*P* = 0.900).

Qualitative analysis of SWE features demonstrated significant differences in the El max of the stiffest portion of the mass of ILC vs IDC. ILC group had significantly higher El max than IDC group (mean value 210.61 ± 35.47 kPa vs 191.64 ± 41.25 kPa, *P* = 0.015) (Table 1). ILC group also had higher El mean than IDC group (180.41 ± 27.96 kPa vs 162.20 ± 37.46 kPa, *P* = 0.008). The differences in El min and in the lesion-to-fat elasticity ratio were not significant (Table 1).

**Table 1:** Elastographic and related sonographic findings in patients with invasive lobular carcinoma (ILC) and invasive ductal carcinoma (IDC)

Characteristics	ILC (N = 40)	IDC (N = 75)
	n (%)	n (%)
Orientation*		
horizontal	30 (75.0)	32 (42.7)
vertical	10 (25.0)	43 (57.3)
Echogenicity*		
hypoechoic	27 (67.5)	69 (92)
hyperechoic	13 (32.5)	6 (8)
Acoustic shadowing		
present	23 (57.5)	44 (58.7)
absent	17 (42.5)	31 (41.3)
Size in cm*^†^	2.25 ± 1.30	1.55 ± 0.90
Maximum elasticity in kilopascals*	210.61 ± 35.47	191.64 ± 41.25
Mean elasticity in kilopascals*	180.41 ± 27.06	162.20 ± 37.46
Minimum elasticity in kilopascals	135.35 ± 32.36	123.48 ± 32.69
Lesion-to-fat ratio of elasticity	8.36 ± 4.19	7.22 ± 3.09

*P < 0.050, χ^2^ test, *t* test.

†mean ± standard deviation.

**Figure 1:**
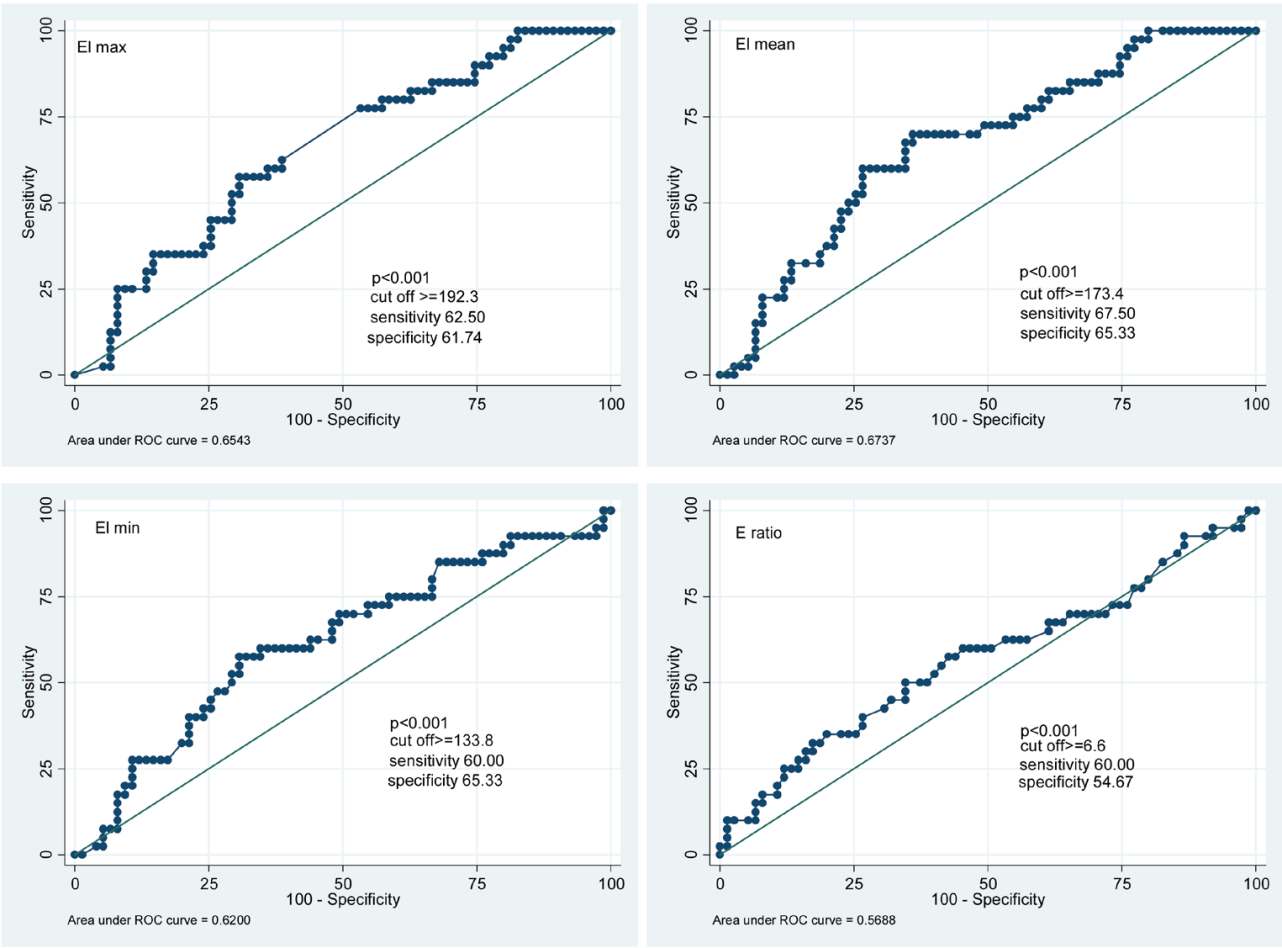
Discriminatory ability of maximum elasticity value of the stiffest portion of mass (El max), mean elasticity value of the stiffest portion of mass (El mean), minimum elasticity value of the stiffest portion of mass (El min), and lesion-to-fat elasticity ratio (E ratio) between invasive lobular breast cancer (ILC) and invasive ductal breast cancers (IDC) patients according to receiver operating characteristic (ROC) curves. Diagnostic efficacy for those values was assessed using the sensitivity and specificity at the specified cut-off point. ROC curve analyses, *P* values <0.050 were considered significant.

The ability of the measured quantitative SWE values to discriminate between IDC and ILC was analyzed using the ROC curve analysis. The two groups could be distinguished on the basis of SWE values of El max (AUC 0.654, 95% CI 0.552-0.757), El mean (AUC 0.674, 95% CI 0.572-0.775), El min (AUC 0.620, 95% CI 0.510-0.729), and E ratio (AUC 0.569, 95% CI 0.454-0.684). Discriminatory ability of El max, El mean, El min, and E ratio between ILC and IDC patients using the sensitivity and specificity at the specified cut-off point according to ROC curves is presented in Figure 1.

22 of 40 (55%) index ILC lesions and 17 of 75 IDC (22.7%) lesions were larger than 2 cm. 15 of 40 (37.5%) ILC lesions and 42 of 75 IDC lesions (56.0%) were between 1-2 cm in size. Only 3 of 40 ILC lesions (7.5%) and 16 of 75 IDC lesions (21.3%) were smaller than 1 cm. If the cut-of value of 1.5 cm is used, 13 of 40 ILC (32.5%) and 47 of 75 IDC (62.7%) lesions were ≤1.5 cm, which represents a significant difference (*P* = 0.002).

Due to the differences in index lesion sonographic size distribution between groups (*P* = 0.008), we stratified our analysis of SWE features by the tumor size (Table 2). Among smaller tumors, with the index lesion size ≤1.5 cm, the El max, El mean, and El min values in ILC group were significantly higher than in IDC group (El max 198.33 ± 36.73 kPa vs 176.22 ± 30.16 kPa, *P* = 0.030; El mean 175.31 ± 31.05 kPa vs 149.44 ± 33.04 kPa, *P* = 0.014; El min 131.23 ± 21.27 kPa vs 113.81 ± 28.38 kPa, *P* = 0.045), while differences in E-ratio were not significant (8.22 vs 6.53, *P* = 0.074). However, in the group of tumors larger than 1.5 cm, no significant differences were observed, and El max, El mean, and El min were even slightly higher in the IDC group.

**Table 2:** Elastographic findings of patients with invasive lobular carcinoma (ILC) and invasive ductal carcinoma (IDC) stratified by sonographic tumor size ≤1.5 cm and >1.5 cm

	ILC≤1.5 cm	IDC≤1.5 cm	ILC>1.5 cm	IDC>1.5 cm
Number of patients (%)	13 (32.5)	47 (62.7)	27 (67.5)	28 (37.3)
Shear-wave elastographic features^†^				
maximum elasticity in kilopascals*	198.33 ± 36.73*	176.22 ± 30.16*	216.53 ± 33.96	218.26 ± 45.45
mean elasticity in kilopascals*	175.31 ± 31.05*	149.44 ± 33.04*	182.86 ± 25.19	183.90 ± 35.67
minimum elasticity in kilopascals*	131.23 ± 21.27*	113.81 ± 28.38*	137.33 ± 36.74	139.46 ± 34.08
lesion-to-fat ratio of elasticity	8.22 ± 4.75	6.53 ± 2.29	8.42 ± 3.99	8.45 ± 3.93

*P < 0.050, *t* test.

†mean ± standard deviation.

All patients with ILC had ER-positive and HER 2-negative cancers, 29 patients (72.5%) had histopathologic grade 2, and 11 patients (27.5%) had grade 3 ILC. 6 patients (15%) had bilateral ILC, and all bilateral cancers were grade 2. Multifocal breast disease was found in 27 patients (67.5%), metastatic axillary lymph nodes in 19 patients (47.5%), and angioinvasion in 14 patients (35%).

Among 13 ILC patients with tumor size ≤1.5 cm (32.5%), multifocal disease was observed on histopatological examination in 8 patients (61.5%), which is almost the same as in 27 patients with ILC index lesion larger than 1.5 cm (70.4%) (19/27). Even though the group of patients with ILC≤1.5 cm was small and consisted of only 13 patients, a significant difference in El max was observed between multifocal and unifocal small tumors (238.1 ± 42.5 kPa vs 180.1 ± 20.1; *P* = 0.011).

**Figure 2:**
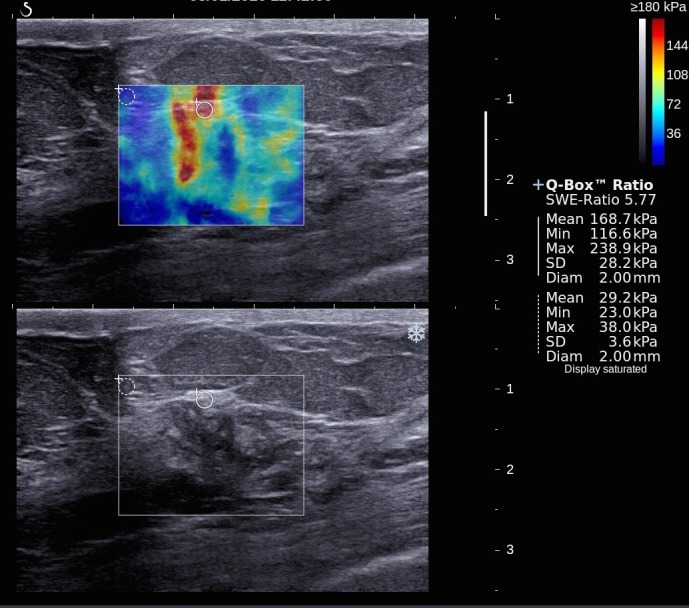
Small, hyperechoic invasive lobular breast cancer (ILC), with high stiffness and high values of El max, El mean, and El min. Abbreviations are explained in Figure 1.

**Figure 3:**
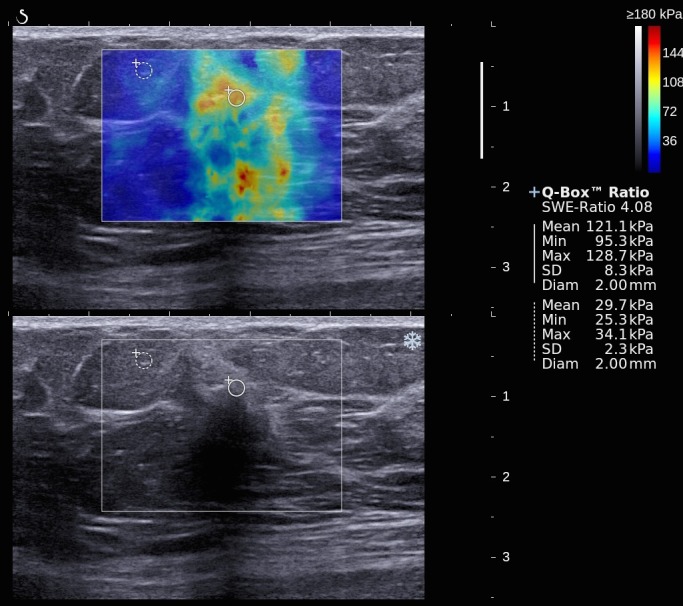
Small, hypoechoic invasive ductal breast cancers (IDC), with low stiffness values of El max, El mean, and El min. Abbreviations are explained in Figure 1.

3/13 patients with small ILC also had axillary lymph node metastases, and the same proportion had angioinvasion (23.1%), while 16/27 patients with larger ILC lesions had axillary metastases (59.3%) and 11/27 had angioinvasion (40.7%). Bilateral disease was found in 6/40 ILC patents (15%); 4 of whom had tumor size ≤1.5 cm. Although ILC patients had higher values in cancers with higher tumor grade, and cancers with axillary metastasis and angioinvasion, no significant difference were found. The examples of the small IDC and small ILC with quantitative SWE measurements are presented in Figures 2 and 3.

## Discussion

It was reported that due to its specific diffusely infiltrating growth pattern ILC was usually larger when detected compared to IDC, and our study is in accordance with these reports (19). The differences in histopathological features are reflected in clinical and imaging manifestations of ILC and IDC (3,5,6,11). We showed differences in sonographic presentation and in sonoelastographic features.-ILC exhibited greater stiffness. However, the difference was pronounced only in tumors with the size ≤1.5 cm, while in larger tumors, which are stiffer than small tumors, SWE stiffness parameters were similar in ILC and IDC.

A study that evaluated the ability of SWE to differentiate between benign and malignant breast tumors found that it improved the specificity of breast ultrasound (20). In a recently published large multicentric study by Berg et al (21), performed in 16 centers in Europe and the USA, the mean El max of 468 invasive cancers, in which IDC and ILC were not separated, and in which the mean diameter of invasive cancer was 15 ± 8.3 mm, was 180 kPa (21). In our study, IDC had higher overall stiffness, with the mean El max of 191.64 kPa but with the slightly larger mean tumor size of 15.5 ± 9.0 mm. When we separately analyzed lesions below the size of 1.5 cm in our study, the stiffness of IDC was 176.22 kPa and of ILC 198.33 kPa. Since the proportion of ILC among invasive cancers encountered in clinical practice is below 10%, our results are not very different compared to the study by Berg et al (21). In the study of Chang et al, 337 invasive breast cancers were evaluated by five radiologists. The mean size of invasive tumor was quite high, 2.2 ± 1.1 cm, and the El mean was 146.8 ± 57.0 kPa. For 16 ILC the El mean was 149.0 ± 51.7 kPa, which was almost the same as for IDC (147.9 ± 57.04 kPa) (25). This study, with much larger cancers than in our or Berg’s study, neither provided El max values nor performed the stratification by size. Therefore, their values cannot be compared precisely to ours. The values of El mean in the Chang’s study were lower than in our study, despite the larger mean tumor size, ie, they were similar to El mean values observed in the group of small IDC in our study, while larger tumors in our study had higher El mean values.

All sonographic examinations, and especially sonoelastographic examinations, are very operator-dependent. In this respect, the advantage of our study was that it was performed by a single experienced examiner on a single high-quality ultrasound scanner, with standardized examination protocol. It was demonstrated, like in other studies, that the size of the measured tumor affected quantitative stiffness values estimated by SWE, so that the larger cancers were stiffer than smaller cancers (21,22,24,25). To the best of our knowledge, our group of patients is the largest group of patients with histopathologically pure ILC analyzed so far with SWE. The fact that ILC lesions observed on ultrasound are significantly larger compared to IDC reflects the clinical practice in which ILC are often found in the advanced stage. Most IDC that we examined were small and were referred to ultrasound after mammography performed in women with no clinical symptoms within the scope of the national mammographic screening program, that is, conducted in the age group of 50-69 years. Mammographic screening allows the diagnosis of small, clinically asymptomatic invasive cancers, which are most often IDC. ILC is often misdiagnosed at mammography in an early stage, shrinking breast mammographic phenomenon may be seen, or the mass is visible only on CC projections (28-30), and the ultrasonographic correlate of mammographically detected lesions is apparently larger, as demonstrated in our study, in which 67.5% of ILC were larger than 1.5 cm, while only 37.3% of IDC were larger than 1.5 cm.

Ultrasonographically measured larger ILC and IDC lesions in our study apparently did not differ in elastographically estimated stiffness. However smaller lesions differed considerably, and small ILC had significantly higher stiffness, which, to the best our knowledge, had not been reported so far. In order to determine why small ILC are stiffer than small IDC we correlated ultrasound findings with the histopathologic findings of resected breast specimens of ILC, since ILC is known to have the propensity for multifocality and bilaterality (3,7,8). Even though the sonographic appearance was of the small mass size, 8 of 13 small ILCs had multifocal disease in the breast on histopathology (61.5%), 4 had bilateral disease, 3 had angioinvasion, and 3 had axillary lymph node metastases. Altogether, 6 of 40 patients (15%) in our patient group had bilateral ILC, which is fewer compared to 20%-29% patients in other studies (7,8). Ultrasonographic measurement of the largest visible mass and elastographic evaluation of stiffness do not seem to be good markers for evaluation of the disease extent in ILC, as is the case in IDC. This is presumably the consequence of the “spiderweb” growth pattern of ILC, which cannot be well estimated by gray-scale ultrasound. Among small ILC, stiffness parameters were higher in multifocal tumors than in unifocal tumors, and although the group is very small the difference in El max reached the level of significance. Therefore, SWE evaluation may be better than gray-scale ultrasound, since it seems to better reflect the disease extent. Studies on larger groups of patients are needed to confirm this observation. It seems reasonable to conclude that ILC with sonographically small index tumor might be larger if they have higher stiffness. This does not happen in the small IDC, which do not exhibit the “spiderweb” growth pattern, but grow as a single focal locally infiltrative mass.

We observed some gray-scale differences between ILC and IDC, which may be useful in daily clinical imaging practice. ILC were significantly more often horizontally oriented than IDC, and had significantly higher proportion of hyperechoic lesions. We observed a low proportion of vertical lesions among ILC (25%), similar to Cawson et al (24%) (31). In our study, ILC and IDC did not differ in the proportion of acoustic shadowing.

Ultrasound is an established imaging modality for detection and differentiation of breast lesions, usually performed to further evaluate suspicious or equivocal mammographic findings and to perform ultrasound-guided biopsy of suspicious breast lesions. The clinical significance of observed differences between ILC and IDC in our study is relatively minor, since all lesions with morphologically suspicious features for invasive cancers need to be subjected to image-guided core biopsy. ILC need to be referred to preoperative contrast-enhanced breast MRI, the best method to assess tumor extent, multifocality, and bilaterality. The presence of some of the described gray scale features and very stiff lesions on SWE may indicate to sonologists that the lesion with morphologic sonographic features of invasive breast cancer might not be the common IDC, but the less common ILC.

The limitation of this study is the relatively small number of patients with ILC. Within the ILC group, we could not demonstrate that stiffness values correlated with histological cancer grade, axillary lymph node involvement, or the presence of angioinvasion, which was demonstrated for IDC in larger patient groups (21). However, it is hard to collect higher numbers of patients with pure ILC in a single center, and it took as five years to collect the group of 40 of patients with all imaging features needed for the study. ILC is a relatively rare type of cancer, seen in fewer than 10% of invasive cancers diagnosed in our institution, and not all patients referred to surgery in our institution were subjected to core biopsy and preoperative ultrasound examination in our department. Another limitation is that the interobsever variability was not studied, since only a single examiner performed all the measurements. However, this setting has ensured the uniform examination technique. Elastographic measurements and analysis are prone to high subjectivity of the examiner. The fact that only the highest values of El max, El mean, and El min were recorded per patient may not reflect the accurate stiffness of lesions, and maybe several measurements in different parts of lesions, with collection of values in different part of tumors, would render higher accuracy. However, the study reflects the usual clinical workflow, in which in a reasonable time frame there needs to be assessed whether a lesion has a low or high stiffness to allow further sonographic management.

As a conclusion, specific histopathologic features of invasive lobular carcinoma seem to be translated into their qualitative sonographic and quantitative sonoelastographic appearance. Sonographically small ILC, with the size ≤1.5 cm, seem to be stiffer than IDC of the same size, while larger tumors exhibit no difference in elastographic parameters. Our results indicate that sonographically small ILC lesions with higher stiffness estimated by SWE-have higher likelihood to be multifocal compared to small ILC lesions with lower stiffness. The knowledge about gray-scale and sonoelastographic features more commonly observed in ILC may help radiologists in diagnosing this relatively uncommon type of invasive cancer, with distinct histopathologic features and different imaging and clinical appearance compared to common IDC.
